# Maintenance of Genetic Diversity Despite Population Fluctuations in the Lesser Prairie‐Chicken (*Tympanuchus pallidicinctus*)

**DOI:** 10.1002/ece3.70879

**Published:** 2025-01-23

**Authors:** Andrew J. Lawrence, Scott A. Carleton, Sara J. Oyler‐McCance, Randy W. DeYoung, Clay T. Nichols, Timothy F. Wright

**Affiliations:** ^1^ Department of Biology New Mexico State University Las Cruces New Mexico USA; ^2^ Division of International Conservation, International Affairs U.S. Fish and Wildlife Service Falls Church Virginia USA; ^3^ U.S. Geological Survey Fort Collins Science Center Fort Collins Colorado USA; ^4^ Caesar Kleberg Wildlife Research Institute Texas A&M University‐Kingsville Kingsville Texas USA; ^5^ Ecological Services United States Fish and Wildlife Service Albuquerque New Mexico USA

**Keywords:** bottleneck, effective population size, genetic diversity, lesser prairie‐chicken, population fluctuations, population structure

## Abstract

Assessments of genetic diversity, structure, history, and effective population size (*N*_e_) are critical for the conservation of imperiled populations. The lesser prairie‐chicken ( *Tympanuchus pallidicinctus* ) has experienced declines due to habitat loss, degradation, and fragmentation in addition to substantial population fluctuations with unknown effects on genetic diversity. Our objectives were to: (i) compare genetic diversity across three temporally discrete sampling periods (2002, 2007–2010, and 2013–2014) that are characterized by low or high population abundance; (ii) examine genetic diversity at lek and lek cluster spatial scales; (ii) identify potential bottlenecks and characterize genetic structure and relatedness; and (iii) estimate the regional *N*_e_. We analyzed 194 samples across the shinnery oak prairie region of eastern New Mexico and western Texas using 13 microsatellite loci. Mean heterozygosity, allelic richness, and inbreeding coefficient were not significantly different between discrete sampling periods, suggesting that this population has maintained its genetic diversity across the sampled population fluctuations. We did not detect genetic structure using multiple Bayesian clustering approaches. Furthermore, there was no support for recent genetic bottlenecks, and we estimated that the *N*_e_ ranged from 229.5 (*p*_crit_ = 0.05, 95% CIs = 121.2–1023.1) to 349.1 (*p*_crit_ = 0.02, 95% CIs = 176.4–2895.2) during our final sampling period (2013–2014). Although we provide evidence for gene flow within this region, continued habitat loss and fragmentation that leads to population declines and isolation could increase the risk of genetic consequences. Continued monitoring of genetic diversity and increasing available habitat that supports robust populations of lesser prairie‐chickens may improve the likelihood of the species' persistence.

## Graphical abstract

Large demographic fluctuations have the potential to alter genetic diversity and structure in wild animal populations. Although lesser prairie‐chicken populations in the southern extent of their range have fluctuated dramatically, this does not appear to have impacted their genetic diversity or structure.

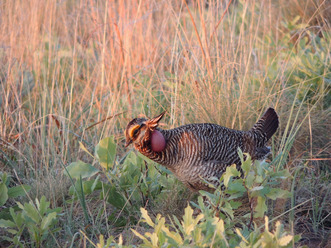

## 1 Introduction

Genetic diversity is a critical component of a species' ability to persist and adapt to environmental change (Lande and Shannon 1996; Frankham 2005). While the contribution of genetic factors to extinction risk has long been a subject for debate, theoretical and empirical studies have shown that populations may be subject to extinction even in the absence of other ecological factors and anthropogenic impacts (Lande 1998; Szűcs et al. 2017). The relationships between population fluctuations, isolation, and genetic diversity remain poorly understood for many species even though they may have significant conservation implications. Small populations can lead to genetic bottlenecks, genetic drift, and inbreeding, which may ultimately lead to extinctions of local populations or even an entire species (Nei, Maruyama, and Chakraborty 1975; Lande 1998; Frankham 2005).

Periodic, high‐amplitude changes in population size are natural for many species and may influence genetic diversity (Elton 1924; Andrewartha and Birch 1954; Potts, Tapper, and Hudson 1984; Hastings and Harrison 1994). The frequency of cycles and amplitude of the fluctuations are largely dependent upon characteristics of the species' life history, resource availability, and other environmental and demographic stochastic events (Ranta, Kaitala, and Lundberg 1998; Bjørnstad and Grenfell 2001). For example, demographic declines of western capercaillie ( *Tetrao urogallus* ), a lekking grouse, were not associated with inbreeding depression, potentially due to increased dispersal of individuals to avoid small leks (Cayuela et al. 2019).

Episodes of substantially reduced population size (*N*) become disproportionately important in determining effective population sizes (*N*_e_) for declining species (Frankham 2005). These fluctuations influence spatial and temporal variation in demographic parameters, which are important for maintaining the genetic diversity of a population (Whitlock 1992). Genetic bottlenecks that occur during population declines have the potential to increase genetic differentiation among populations, decrease genetic diversity within a population, and increase the possibility of inbreeding depression (Nei, Maruyama, and Chakraborty 1975; Motro and Thomson 1982; Gyllensten 1985; Frankham 1995, 2005; Keller and Waller 2002), which, in turn, can increase extinction risk (Frankel and Soulé 1981).

Although basic population genetics theory suggests that genetic diversity should decrease with decreases in *N*_e_, enhanced gene flow associated with local extirpation and recolonization can limit differentiation among populations and mitigate the degenerative effects of population crashes (Nei, Maruyama, and Chakraborty 1975; Slatkin 1977; Berthier et al. 2006). Increased dispersal during periods of high population density may mitigate mate competition, inbreeding, and pathogen pressure and improve access to resources (Travis, Murrell, and Dytham 1999; Smith et al. 2009; Lutz, Diefenbach, and Rosenberry 2015). Negative density‐dependent dispersal may also accelerate gene flow and compensate for genetic drift during population crashes (Ims and Andreassen 2005). Under these conditions, dispersal increases during crash phases, which could be the result of scarcity of mates and inbreeding avoidance (Pusey and Wolf 1996; Andreassen and Ims 2001; Ims and Andreassen 2005). Genetic diversity of recovered populations may be relatively high if the new population is founded by a mixture of individuals from different areas (Mills and Allendorf 1996), but there may be increased risk of inbreeding depression if a population is founded by individuals from just one source (Nei, Maruyama, and Chakraborty 1975; Szűcs et al. 2017). High levels of migration can also affect the magnitude of genetic change and synchronize the genetic responses of geographically separated populations (Whitlock 1992; Franklin, Myers, and Cory 2014). Overall, the effects of population fluctuations on genetic diversity are diverse, potentially substantial, and may be difficult to predict a priori.

The lesser prairie‐chicken ( *Tympanuchus pallidicinctus* ) is an imperiled grassland grouse whose genetic diversity may be influenced by population fluctuations, especially given its substantial population declines. Similar to other gallinaceous birds, lesser prairie‐chicken populations experience “boom and bust” (i.e., high vs. low abundance) years driven by variation in local precipitation patterns, but it has also experienced a long‐term decline over the past century (Hagen and Giesen 2020). Historical (pre‐1900) population estimates are limited by data collection standards of the time, but the species' estimated range has decreased by 78% since 1963 (Hagen and Giesen 2020). Although no single factor has been solely responsible for lesser prairie‐chicken population declines, habitat loss and fragmentation, and the species' response to novel (i.e., within recent decades) anthropogenic infrastructure and invasive trees and shrubs are considered to be important contributors (Taylor 1980; Hagen and Giesen 2020; Lawrence et al. 2021; USFWS 2021). These substantial declines, punctuated by cycles of high and low abundance, also have the potential to cause population bottlenecks with genetic consequences (Nei, Maruyama, and Chakraborty 1975; Bouzat et al. 1998).

Population variability for lesser prairie‐chickens is reflected in the abundance of individuals attending leks (i.e., mating grounds) in the spring, and the number of active leks in an area is positively correlated with the density of males attending leks (Cannon and Knopf 1981). Lek attendance is highly variable, such that declines, collapses (i.e., no attending birds), and recolonizations have been frequently observed but are poorly understood (Garton et al. 2016; Behney et al. 2012). Some leks are consistently attended for several decades (Copelin 1963), while others are transient and experience higher turnover rates (Hagen and Giesen 2020). Lek stability is likely influenced by the number and age of males, lek density, and environmental factors like precipitation, with anthropogenic declines further exacerbating these effects (Schroeder and Braun 1992; Haukos and Smith 1999; Hagen and Giesen 2020).

As part of their lek mating system, males gather to compete for mates by performing elaborate vocal and visual courtship displays (Sharpe 1968; Hagen and Giesen 2020). Males that display more frequently and for longer bouts are more likely to be selected by females for copulation, commonly resulting in highly skewed mating success rates for relatively few males within a lek (Wiley 1974; Behney et al. 2012). In one study, a single male lesser prairie‐chicken was responsible for 79% of the copulation attempts at one lek (Behney et al. 2012). Because of this strong sexual selection and skewed mating success, lesser prairie‐chickens are predicted to have a relatively low *N*_e_ (Bouzat and Johnson 2004; Pruett et al. 2011). The genetic diversity of lesser prairie‐chicken populations may be further reduced when individuals within a subpopulation are closely related; however, strong differentiation among subpopulations, should it exist, could maintain more genetic diversity at the population level (Bouzat and Johnson 2004).

Despite the fact that cyclic population fluctuations are common for gallinaceous birds and are well documented for lesser prairie‐chickens, the effects of such patterns on genetic diversity and structure are rarely described, even though they may have important conservation implications (DeYoung and Williford 2016). To address this knowledge gap, we examined genetic diversity and structure for the southernmost populations of lesser prairie‐chickens in New Mexico and Texas, USA. Our objectives were to examine the genetic diversity of fluctuating populations of lesser prairie‐chickens in a landscape fragmented by anthropogenic development and woody vegetation encroachment. Specifically, we examined the lesser prairie‐chicken: (i) genetic diversity over time (2002, 2007–2010, and 2013–2014) at varying population abundances and at lek and lek cluster spatial scales; (ii) population history (i.e., bottlenecks), relatedness, and population structure; and (iii) the regional genetic effective population size (*N*_e_). We conducted these analyses for three discrete sampling periods that occurred in 2002, 2007 to 2010, and 2013 to 2014, with greater focus on the final sampling period as it is most relevant to current conditions.

## 2 Methods

**FIGURE 1:**
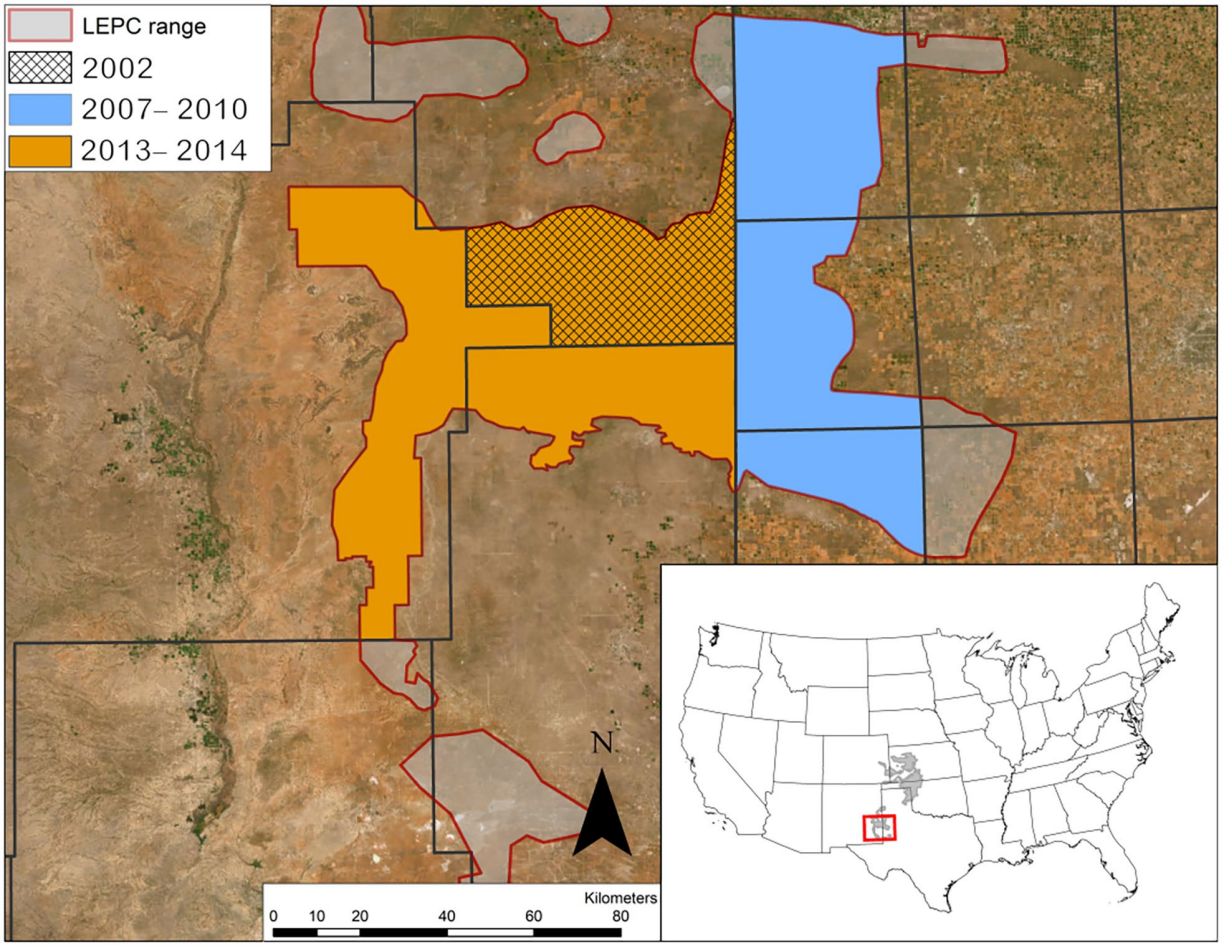
Sampling locations for analyses of lesser prairie‐chicken (LEPC) genetic diversity changes between 2002, 2007–2010, and 2013–2014. Chaves, Lea, and Roosevelt Counties, New Mexico, are shown in orange, while Bailey, Cochran, and Yoakum Counties, Texas, USA are shown in blue.

We collected blood, tissue, or feather samples across three discrete sampling periods: in 2002 (in Roosevelt County, New Mexico), in 2007 to 2010 (in Bailey, Cochran, and Yoakum Counties, Texas), and in 2013 to 2014 (in Chaves, Lea, and Roosevelt Counties, New Mexico; Figure 1). Sample size varied among collection sites due to differences in the number of individuals available for capture and sampling effort, which was lower for leks that were visited primarily for the collection of molted feathers that had no direct capture effort (Table 1). All samples were confirmed as having unique multilocus genotypes. Analyses for 2013–2014 focused on approximately 2840 km^2^ in Chaves, Lea, and Roosevelt counties, New Mexico (Figure S1). We considered these sampling periods to be discrete because the time between them exceeds the average lifespan (< 2 years) of a lesser prairie‐chicken (Haukos and Boal 2016). Our assumption that the three sampling periods constitute a single population is based on the results of Oyler‐McCance et al. (2016), which are supported by additional analyses in this study (refer to Section 2.7).

**TABLE 1:** Mean observed heterozygosity (*H*_O_) and expected heterozygosity (*H*_e_), mean allelic richness across loci (*AR*), and total number sampled individuals and private alleles for each lek cluster in 2013–2014 in Chaves, Lea, and Roosevelt Counties, New Mexico, USA.

Locality	Mean *H*_O_	Mean *H*_e_	Mean *AR*	Private alleles	Sample size
1	0.66 (0.38)	0.70 (0.31)	1.71 (0.27)	—	2
2	0.79 (0.23)	0.72 (0.18)	1.73 (0.18)	1	4
3	0.73 (0.14)	0.78 (0.08)	1.78 (0.08)	—	14
4	0.80 (0.19)	0.77 (0.12)	1.77 (0.12)	—	9
5	0.76 (0.18)	0.75 (0.16)	1.76 (0.16)	3	10
6	0.78 (0.30)	0.73 (0.26)	1.74 (0.26)	—	5
7	0.70 (0.33)	0.70 (0.23)	1.71 (0.16)	—	2
8	0.76 (0.12)	0.74 (0.11)	1.74 (0.11)	1	10
9	0.61 (0.37)	0.73 (0.22)	1.66 (0.28)	—	3
10	0.77 (0.16)	0.76 (0.11)	1.77 (0.11)	7	22
11	0.65 (0.26)	0.78 (0.12)	1.76 (0.11)	—	4
Overall	0.75 (0.12)	0.76 (0.11)	8.19 (3.49)	—	85

*Note:* We report the standard deviation in parentheses. Overall mean *H*_O_, *H*_e_, and *AR* were calculated for the entire sampled population.

### 2.1 Study Area

Lesser prairie‐chickens in our study area are primarily restricted to the shinnery oak ( *Quercus havardii* ) prairie of the Llano Estacado and surrounding southern Great Plains. The shinnery oak prairie community was the dominant community in this region, and common grasses included dropseeds (*Sporobolus* spp.), little bluestem ( *Schizachyrium scoparium* ), and gramas (*Bouteloua* spp.). Shinnery oak was the most common shrub, followed in abundance by sand sagebrush ( *Artemisia filifolia* ) and honey mesquite ( *Prosopis glandulosa* ). An introduced Asian tree, Siberian elm ( *Ulmus pumila* ), was occasionally found along roadsides and sites of current or former human habitation. Honey mesquite and Siberian elm are avoided by lesser prairie‐chickens and reduce the quality of remaining habitat (Boggie et al. 2017; Lawrence et al. 2022). Much of the species' current and historic range in this region overlaps with the Permian Basin, one of the most productive oil and natural gas regions in the United States (Independent Petroleum Association of New Mexico 2016). Many features associated with anthropogenic development are avoided by lesser prairie‐chickens (Hagen et al. 2011; Plumb et al. 2019; Lawrence et al. 2022) and may have the potential to limit gene flow between populations as has been documented for other species (Hutchison and Templeton 1999; Cegelski, Waits, and Anderson 2003; Epps et al. 2005; Yang and Jiang 2011). Varying densities of anthropogenic features (e.g., oil well pads, utility poles) were interspersed throughout our study areas, including one highway (U.S. Route 380) that appears to restrict movements of lesser prairie‐chickens between leks to the south and north of the highway (Figure S1; Lawrence et al. 2022).

### 2.2 Sample Collection

The majority of DNA samples were feathers plucked from individuals captured at leks from March through late May using walk‐in funnel traps, drop nets, and whoosh nets (Haukos, Smith, and Broda 1990; Coxen, Collins, and Carleton 2018; Lawrence et al. 2021). We also collected molted feathers (primarily at lek sites), eggshell remains from nests, and muscle tissue from recently deceased individuals (e.g., roadkill, predator mortalities). Each feather collection from a live bird consisted of 4–5 contour feathers, which we stored in paper coin envelopes at room temperature in a 29 L weather‐resistant container with two 200 g silica gel desiccant bags. Blood samples were collected from the brachial vein or clipped toenail of captured individuals, stored in microfuge tubes coated with EDTA, and kept frozen at −20°C until DNA extraction. Capture and handling were permitted by the New Mexico Department of Game and Fish and performed according to New Mexico State University Institutional Animal Care and Use Committee protocol number (#2013‐015).

### 2.3 DNA Extraction and Amplification

We extracted DNA from the feather samples using an established protocol for the purification of DNA from nails, hair, or feathers with the DNeasy 96 Blood and Tissue Kit (Qiagen). This protocol was slightly modified in the elution step, namely we eluted in 100 μL of Buffer AE after incubation for 5 min at room temperature. We genotyped individuals using 13 microsatellite loci: MSP11, SGMS06.2, SGMS06.3 (Oyler‐McCance and St. John 2010), TUT3, TUD1 (Segelbacher et al. 2000), BG6, BG18 (Piertney and Höglund 2001), TTT1, TTD6 (Caizergues et al. 2001), ADL230 (Cheng et al. 1995), SG24, SG27, SG31 (Fike et al. 2015), and sexed each sample using the primers 1237L and 1272H (Kahn, St. John, and Quinn 1998). These methods are also described in detail by Oyler‐McCance et al. (2016). All datasets used in our analyses were genotyped in the same laboratory using the same loci and scoring methods.

### 2.4 Lek Trend Data

Lesser prairie‐chickens have experienced substantial declines with incomplete recoveries in recent decades, and leks in our study area in New Mexico followed previously observed boom and bust trends for the species. We characterized lek attendance trends (i.e., the number of individual lesser prairie‐chickens present at a lek over time) and compared these data with measures of genetic diversity across sampling periods. Lek data were collected during our study and were provided by the Bureau of Land Management, Natural Heritage New Mexico, and New Mexico Department of Game and Fish (survey methods in Beauprez 2011). We used the highest observed count of individuals for leks that were surveyed multiple times within 1 year from 2000 and 2014. We selected this time period as it encompassed the entire period of our genetic sampling and contained substantial changes in lek attendance.

We characterized measures of genetic diversity for 2013–2014 at two spatial scales: lek clusters and individual leks. By repeating our analyses at these two scales, we address the potential confounding effects of spatial scales on gene flow and genetic variation. We defined lek clusters as groups of leks that occurred within 6 km of each other, or leks that were linked by 6 km “stepping‐stone” distances (Figure S1). In New Mexico, more than 95% of movements from the lek at which lesser prairie‐chickens were captured fall within this radius (Lawrence et al. 2022). So, leks > 6 km apart could still be part of the same group if another intermediate lek between them was < 6 km away.

Associating population fluctuations with potential genetic responses requires consideration for the species' life history and appropriate representations of population trends. We used a generation length of 1.5 years based on age at first reproduction (1 year) with an estimated annual survival rate based on previous studies (0.33 averaged across both sexes, Meyers et al. 2018). We used the generation length (*T*) formula where *T* = *M* − 1 + *A* (Pruett et al. 2011), where *M* is the time that first breeding occurs (first year for lesser prairie‐chickens, Hagen and Giesen 2020). The value for *A* is calculated using the formula *A* = 1/(1 − *V*), where *V* is the average survival rate of lesser prairie‐chickens (based on Meyers et al. 2018). We multiplied this generation time by 10 (estimated number of generations for genetic response to register; Leblois, Rousset, and Estoup 2004) to warrant the selection of 15 years to examine genetic data. The genetic diversity of species with a naturally low *N*_e_ that are experiencing low densities and large population fluctuations, such as the lesser prairie‐chicken, can be shaped by both present and recent demographic changes (i.e., less than 10 generations; Leblois, Rousset, and Estoup 2004). To compare genetic diversity levels between sampling periods, we used either Wilcoxon signed‐rank tests for mean observed heterozygosity (*H*_O_) and *t*‐tests for mean allelic richness (AR) and mean *F*_IS_ (inbreeding coefficient) after testing for normality.

### 2.5 Population Genetics Analyses

We checked loci for null alleles, linkage disequilibrium, and deviations from Hardy–Weinberg proportions and verified that all samples represented unique individuals using the R (v 3.4.4, 2018) package *PopGenReport* v 3.0 (Adamack and Gruber 2014) and GENEPOP v 4.2 (Rousset 2017). The *null.all* function in *PopGenReport* checks for null alleles and estimates bootstrap confidence intervals for each locus using the methods of Chakraborty et al. (1994) and Brookfield (1996), while GENEPOP's estimate uses only the Brookfield (1996) method. We removed loci that presented null alleles with frequencies significantly higher than zero prior to subsequent analyses. Using GENEPOP, we tested for linkage disequilibrium for each pair of loci in each sampling period using the log likelihood ratio with Markov chain parameters set at 10,000 dememorization, 1000 batches, and 10,000 iterations per batch. Linkage disequilibrium significance values were adjusted using the Bonferoni correction (*α*/*n*; *n* = 66 pairwise loci comparisons). We also assessed the statistical significance of linkage disequilibrium tests by determining the proportion of tests that were statistically significant for each sampling period. A proportion lower than the Type I error rate would suggest no evidence for linkage disequilibrium (Waples 2015). We did not find support for loci with a significant probability of linkage disequilibrium or a significant deviation from Hardy–Weinberg proportions, and thus did not remove additional loci from the dataset. We calculated observed (*H*_O_) and expected heterozygosity, the numbers of alleles per locus and private alleles, *AR*, *F*_IS_, and pairwise *F*_ST_ (genetic variation in a subpopulation relative to the total population; calculated according to Weir and Cockerham 1984) among sampling locations using the *hierfstat* v. 0.5‐11 package (Goudet and Jombart 2022) in program R v 3.4.4 (R Core Team 2018). We also compared the number of private alleles, mean *H*_O_, *AR*, and *F*_IS_ among sampling periods.

To examine pairwise relatedness, *r*, and relationships between individuals within each sampling period, we used the program ML‐Relate (Kalinowski, Wagner, and Taper 2006). We tested for differences in *r* between sampling periods with a Wilcoxon signed‐rank test and estimated the most likely relationship between all pairs of individuals with 95% confidence intervals using 10,000 repetitions.

### 2.6 Population History and Effective Population Size

To evaluate whether the lesser prairie‐chicken population for each sampling period of our study has experienced a recent genetic bottleneck, we conducted a mode‐shift analysis to detect change of the expected L‐shaped distribution of allele frequency (Cornuet and Luikart 1996). We also estimated heterozygosity excess or deficit using the null hypothesis of mutation‐drift equilibrium under the two‐phase mutation (TPM) model in BOTTLENECK 1.2.02 (Piry, Luikart, and Cornuet 1999). The TPM is considered better suited at accounting for the mutational dynamics at microsatellite loci than alternative stepwise mutation models (Di Rienzo et al. 1994; Piry, Luikart, and Cornuet 1999). We ran TPMs using 60% and 80% stepwise mutations for 10,000 iterations with variance set to 12, as suggested by Piry, Luikart, and Cornuet (1999). Approximately 60%–80% of avian microsatellite mutational dynamics involve a single‐step change (Brohede et al. 2002; Brohede, Møller, and Ellegren 2004; Beck, Double, and Cockburn 2003; Ortego et al. 2008), thus we chose to bracket TPM analyses with those values. We conducted the Sign test and Wilcoxon signed‐rank test to evaluate significant heterozygosity excess (Piry, Luikart, and Cornuet 1999).

To estimate effective population size (*N*_e_) for each sampling period, we used NeEstimator v 2.01 (Do et al. 2014) using linkage disequilibrium methods (Waples 2006; Waples and Do 2010; Waples, Antao, and Luikart 2014) and pooled individuals across years but still within discrete sampling periods. Inclusion of rare alleles can upwardly bias linkage disequilibrium‐based estimates, so we used allele frequency restrictions (*p*_crit_) of 0.02 or greater (Waples and Do 2010). We derived 95% confidence intervals (CIs) using the jackknife option, which is a more conservative option if locus pairs are not entirely independent (Waples 2006; Do et al. 2014).

### 2.7 Clustering Analyses

Bayesian clustering algorithms are frequently used to evaluate genetic population structure (Beaumont and Rannala 2004), and using several methods to improve confidence is increasingly common (Campos et al. 2021; Oyler‐McCance et al. 2022). We applied multiple Bayesian clustering approaches to evaluate the relationships between individuals and provenances. We used STRUCTURE v 2.3.4 (Pritchard, Stephens, and Donnelly 2000) to examine population structure within and among sampling periods. We ran STRUCTURE without population origin or spatial coordinates as prior, with *K* ranging from 1 to 10. We used an admixture model with correlated allele frequencies, suitable for individuals with admixed ancestry (Pritchard, Stephens, and Donnelly 2000). Each *K* value had 10 independent runs, with 500,000 MCMC (Markov chain Monte Carlo) iterations, discarding the first 500,000 as burn‐in. Following Wang (2017), we also ran STRUCTURE to account for unbalanced sample sizes by setting alpha as 0.33 (1 divided by 3, i.e., the three sampling periods), but this did not appear to improve the results (data not shown). The results and visual output of the 10 iterations for each *K* value were summarized using CLUMPAK (Kopelman et al. 2015). We determined the *K* value that best explained the data with the log likelihood of each *K* (ln*L*) (Pritchard, Stephens, and Donnelly 2000) and with the parameter Δ*Κ* (Evanno, Regnaut, and Goudet 2005).

We used the *Geneland* v 4.0.8 (Guillot, Mortier, and Estoup 2005) package in R as an additional estimator of population structure. We conducted two independent analyses using correlated and uncorrelated allele frequency models to evaluate the optimal value of *K*. Each run had 100,000 iterations, 100 burn‐in, thinning of 100, and an uncertainty of spatial coordinates set to 6 km. Although we had exact locations of sample collections, movement data for lesser prairie‐chickens in eastern New Mexico have revealed that > 95% of an individual's locations are within 6 km of the lek of capture (Lawrence et al. 2022). Thus, a 6 km uncertainty for spatial coordinates should help account for potential movement of individuals among closely spaced leks. We determined the optimal value of *K* by examining the posterior probabilities averaged over multiple runs (ten runs allowing *K* to vary from 1 to 10) and choosing the *K* value with the highest average posterior probability.

## 3 Results

### 3.1 Sample Acquisition and Amplification

We collected 48, 62, and 86 samples in 2002 (Pruett et al. 2011), 2007–2010 (Oyler‐McCance et al. 2016), and 2013–2014, respectively. Seventy‐six samples from 2013 to 2014 were plucked feathers from captured individuals, and the remaining 10 were collected as molted feathers or from other tissues found opportunistically in the field at 27 locations (Figure S1). For our final sampling period, all but one sample were collected in 2014.

We removed two individuals from subsequent analyses that were missing data at three or more loci; the remaining individuals were missing data at two or fewer loci. Both GENEPOP and *PopGenReport* identified locus BG6 as having a high estimated frequency of null alleles (0.6) in 2013–2014; thus, we removed BG6 from subsequent analyses. Although the samples from the first two sampling periods did not show evidence of null alleles, we used the same suite of loci for comparisons of genetic diversity among sampling periods. No other locus deviated from Hardy–Weinberg equilibrium (HWE) or showed detectable linkage disequilibrium, leaving 12 remaining loci for further analyses. We observed a total of 104 alleles across all loci, and the number of alleles per locus ranged from 4 to 16 in 2013–2014 (Table 2).

**TABLE 2:** Observed heterozygosity (*H*_O_), expected heterozygosity (*H*_e_), number of alleles (*n*), and locus‐specific genetic differentiation (*F*_ST_) and *F*_IS_ values for microsatellite loci of lesser prairie‐chickens in 2013–2014 in Chaves, Lea, and Roosevelt Counties, New Mexico, USA.

Locus	*H*_O_	*H*_e_	*n*	*F*_ST_	*F*_IS_
BG18	0.84	0.81	7	0.02	−0.03
MSP11	0.77	0.89	15	−0.08	0.14
SGMS062	0.79	0.72	8	0.07	−0.10
TTD6	0.74	0.75	8	0.08	0.01
TTT1	0.73	0.84	8	−0.10	0.14
ADL230	0.75	0.80	9	0.03	0.07
SG24	0.87	0.89	16	−0.03	0.02
SG31m13	0.71	0.67	6	0.04	−0.04
TUD1	0.91	0.89	12	−0.01	−0.02
SG27m13	0.50	0.49	4	0.01	0.00
SGMS063	0.75	0.67	5	−0.02	−0.11
TUT3	0.66	0.66	6	0.02	0.01

### 3.2 Population Genetics Analyses

Observed heterozygosity (*H*_O_) across loci for all sampled individuals ranged from 0.5 to 0.91 (Table 2) in 2013–2014. The mean *H*_O_ per locus did not vary significantly among loci, and a Bartlett test of homogeneity of variances for the observed and expected heterozygosity was not significant (*p* = 0.69). For lek clusters, mean *H*_O_ and mean *AR* ranged from 0.61 to 0.8 and from 1.66 to 1.78, respectively (Table 1). There were four lek clusters with private alleles (range = 1–7). Mean *H*_O_ per lek ranged from 0.58 to 1.0, while mean *AR* ranged from 1.58 to 2.0 for lek sites and was 8.19 for the total sampled population. Several leks were each represented by one individual, so we report the observed values for that lek in lieu of a mean. Six leks had at least one private allele (range = 1–4, Table S2).

Our relatedness analyses estimated low and similar mean levels of relatedness, *r*, within each sampling period. The mean *r* was 0.052 (SE = 0.002, range = 0–0.64), 0.052 (SE = 0.001, range = 0–0.58), and 0.053 (SE = 0.001, range = 0–0.75) for sampling periods 1, 2, and 3, respectively (Supporting Information).

### 3.3 Population History and Effective Population Size

There was mixed evidence for a recent genetic bottleneck for lesser prairie‐chickens for each sampling period. Significant heterozygosity excess was detected by one‐tailed Wilcoxon tests for the 60% stepwise mutation level for 2007–2010 and 2013–2014, but for both stepwise mutation levels only in 2013–2014. Only the 60% test in the final sampling period supported a genetic bottleneck with the Sign test (*p* = 0.01). The mode‐shift analysis indicated normal L‐shaped distributions of microsatellite allele frequencies for each sampling period, providing further support for the lack of genetic bottlenecks. The estimated *N*_e_ varied among sampling periods (Table 3), which may reflect both actual population fluctuations and differences in sampling effort between sampling periods. The *N*_e_ for 2013–2014 was below the estimated population size for the shinnery oak prairie region in 2014 (*N* = 1155) when the majority of samples were collected in our final sampling period.

**TABLE 3:** The number of private alleles (*PA*), mean observed heterozygosity (*H*_O_), mean expected heterozygosity (*H*_e_), mean allelic richness (*AR*), mean inbreeding coefficient (*F*_IS_), estimated genetic effective population size (*N*_e_) using the 0.05 allele frequency level, and total number of individuals (*n*) for each sampling period in Chaves, Lea, and Roosevelt Counties, New Mexico, USA, and in Bailey, Cochran, and Yoakum Counties, Texas, USA.

Sampling period	*PA*	Mean *H*_O_	Mean *H*_e_	Mean *AR*	Mean *F*_IS_	*N*_e_	*n*
2002	8	0.74 (0.11)	0.74 (0.12)	8.04 (3.10)	0.002 (0.054)	76.3 (36.2–470.4)	48
2007–2010	8	0.73 (0.11)	0.75 (0.11)	7.93 (3.34)	0.028 (0.074)	255.7 (95.7–∞)	61
2013–2014	14	0.75 (0.12)	0.76 (0.11)	8.19 (3.49)	0.012 (0.058)	229.5 (121.2–1023.1)	85

*Note:* The number of private alleles were identified for each sampling period, and the mean *H*_O_, *H*_e_, and *AR* were calculated across all loci. We report the standard deviation for each mean, as well as the confidence intervals for *N*_e_, in parentheses. A Wilcoxon signed‐rank test for *H*_O_ and *t*‐tests for *AR* and *F*_IS_ did not detect significant pairwise differences of these values among sampling periods.

### 3.4 Lek Population Trends and Genetic Diversity

**FIGURE 2:**
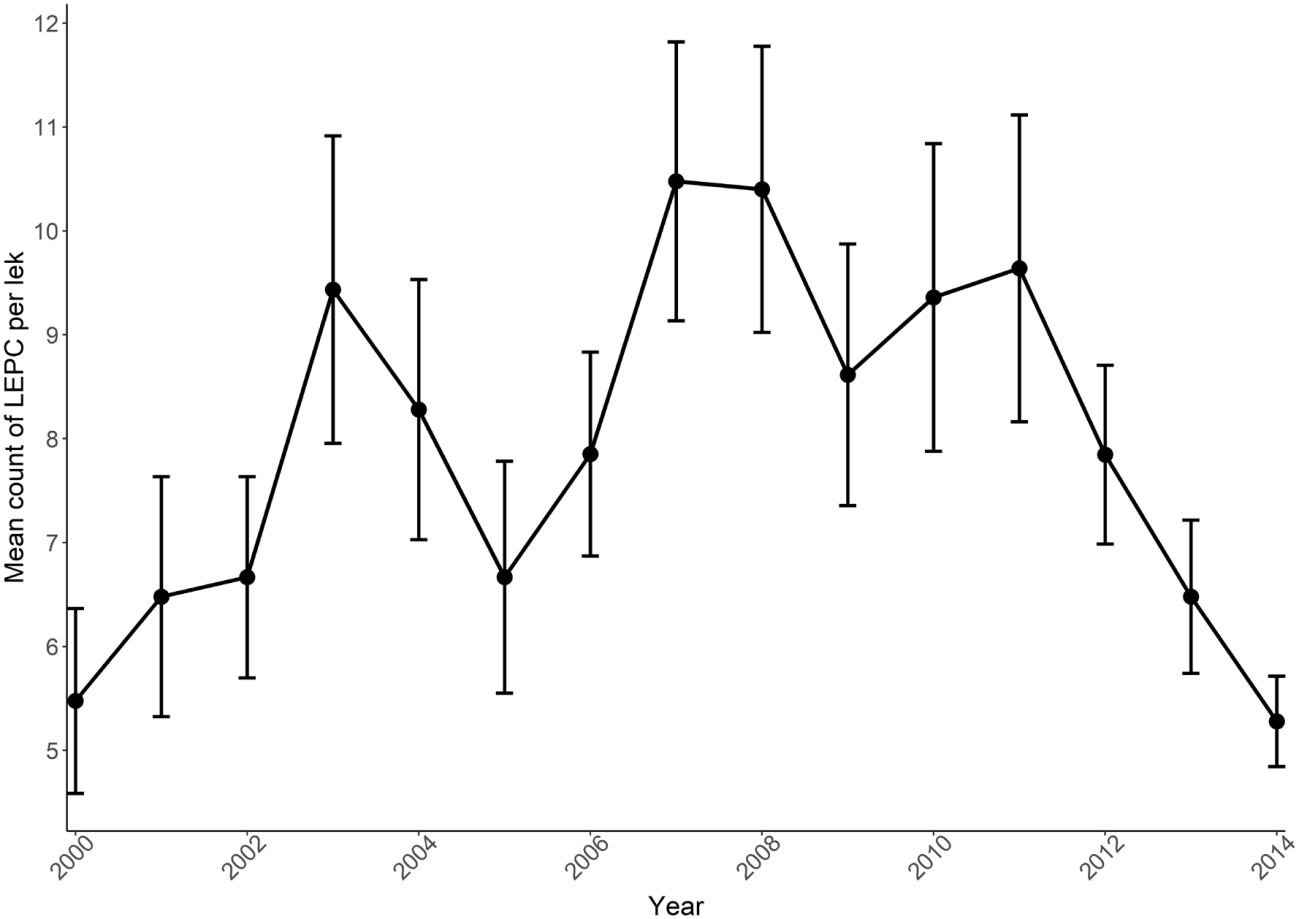
Patterns of lesser prairie‐chicken (LEPC) attendance across sample sites in Chaves, Lea, and Roosevelt Counties, New Mexico, USA from 2000 to 2014. Counts represent the mean maximum number of lesser prairie‐chickens, including standard errors, observed across leks in our study within a given year. Mean counts for all leks across years ranged from 5.2 (SE = 0.4) in 2014 to 10.5 (SE = 1.3) in 2007.

Lesser prairie‐chicken populations in our study area were substantially reduced during 2002 and 2013–2014 and grew to their peak during 2007–2010 (Figure 2). The mean count of individuals attending these leks grew from 5.4 (SE = 0.96) in 2000, peaked at 10.5 (SE = 1.34) in 2007, and remained relatively high until declining dramatically to 5.2 (SE = 0.43) in 2014. The mean lek‐to‐nearest‐lek distance in our final sampling period (2013–2014) was 4.1 ± 0.7 km and ranged from 1.3 to 18.7 km. Although our measures of genetic diversity may have changed with these population trends, we did not find statistically significant pairwise differences in mean *H*_O_, mean *AR*, or mean *F*_IS_ between the sampling periods (Table 3).

### 3.5 Population Structure

**FIGURE 3:**
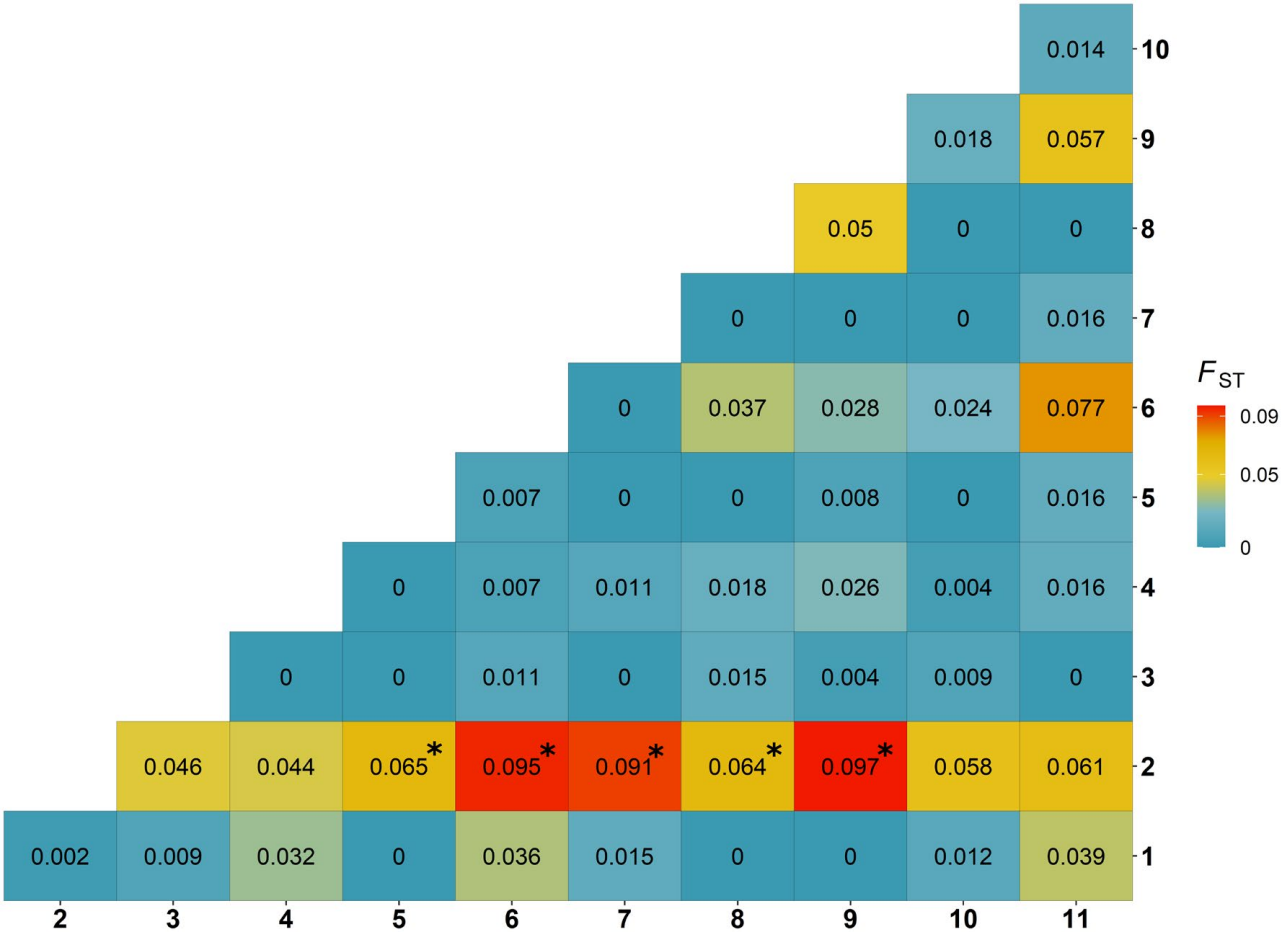
Pairwise population matrix of *F*_ST_ values (Weir and Cockerham 1984) for lesser prairie‐chicken lek clusters in Chaves, Lea, and Roosevelt Counties, New Mexico, USA in 2013 and 2014. Pairs with values significantly greater than zero (*p* < 0.05) are denoted with asterisks. Corresponding lek cluster identification numbers are available in Figure S1.

We did not find evidence for population differentiation for lesser prairie‐chickens in our study area or between sampling periods, including when adjusting for unbalanced sample sizes among sampling periods. The *K* with the highest likelihood based on STRUCTURE output was *K* = 1 (Figure S3) when we evaluated all three sampling periods together. The optimal Δ*Κ* from the STRUCTURE analysis using the Evanno method estimated *K* = 2 (Table S1; Figure S4), but this method cannot find the best *K* if *K* = 1 (Evanno, Regnaut, and Goudet 2005; Janes et al. 2017). Bayesian clustering analyses using Geneland did not find evidence for *K* > 1 using either correlated or uncorrelated allele models (Figure S5). Pairwise *F*_ST_ values estimated according to Weir and Cockerham (1984) were low for all lek combinations in 2013–2014, except for lek cluster 2, which was the most divergent relative to other lek clusters (Figure 3). Lek 2, which was included in lek cluster 2, was similarly divergent from other leks and had *F*_ST_ significantly > 0 for five other leks (Figure S2). Lek cluster 2 and several others had low sample sizes, however, which reduces our confidence in these results.

## 4 Discussion

Studies of genetic diversity and the factors that influence it are important contributions to the conservation of threatened species. We collected genetic samples from lesser prairie‐chickens across southern portions of the species' range in the shinnery oak prairie ecoregion and generated a microsatellite dataset to examine patterns of genetic diversity and structure, population history, the influence of cyclic populations on genetic diversity, and to estimate the effective population size. Overall, we found relatively high genetic diversity and a lack of genetic structure, despite evidence for recent population fluctuations. The apparent lack of a genetic bottleneck was supported by multiple analytical and comparative analyses.

While our analyses did not identify genetically isolated subpopulations, it is important to consider that the severity and duration of population isolation and decline may not have been sufficient to produce a detectable genetic response (Landguth et al. 2010). Lesser prairie‐chicken populations in New Mexico have declined dramatically since the late 1980s and have not fully recovered since (Bouzat and Johnson 2004). From 1998 to 2008, the minimum population size based on lek counts in New Mexico grew from < 1000 to > 9000 individuals (G. Beauprez, New Mexico Department of Game and Fish, written communication, 2017), before declining again to approximately 1155 individuals (90% CIs = 337–2210) in 2014 (Nasman et al. 2022). The time lag for genetic effects to register from these events and the potential for interactive effects of anthropogenic impacts are uncertain.

### 4.1 Population Genetics Analyses

Some measures of genetic diversity for our study contrasted with those of previous studies of grouse genetic diversity (Table 4), while others were similar. The mean *H*_O_ for our study (mean *H*_O_ = 0.75) was higher than what has been previously reported for lesser prairie‐chickens in New Mexico, for which mean *H*_O_ ranged from 0.4 to 0.583 (Van Den Bussche et al. 2003) and 0.53–0.548 (Bouzat and Johnson 2004). Both of these studies reported genetic differentiation based on smaller sample sizes (*n* = 44 and 66, respectively) and at smaller geographic scales (sample site distances ranged 1–19 km apart). Our study used approximately twice the number of microsatellite loci, in addition to an updated suite of loci that is more specific to grouse. The confounding effects of ascertainment bias on measures of genetic diversity may limit the comparability of our data with studies that used loci from more distantly related species (Wright et al. 2004).

**TABLE 4:** Comparative summary of select studies that used microsatellites to examine patterns of grouse genetic diversity and effective population size.

Species	Region	# of Loci	AR	*H*_O_	*N*_e_	Reference
*Tympanuchus pallidicinctus*	NM	12	8.19	0.75	229.5–349.1	Present study
*Tympanuchus pallidicinctus*	SOPR	13	7.83	0.71	252	Oyler‐McCance et al. (2016)
*Tympanuchus pallidicinctus*	NM	6	4.33–5.83	0.53–0.54	NA	Bouzat and Johnson (2004)
*Tympanuchus pallidicinctus*	NM; OK	5	*A* = 2.4–4.8 (NM), 2.2–5.8 (OK)	0.4–0.58	NA	Van Den Bussche et al. (2003)
*Tympanuchus pallidicinctus*	NM; OK	8		NA	54.9–57.6 (NM), 69.4–114.7 (OK)	Pruett et al. (2011)
*Tympanuchus cupido*	Midwestern USA	6	*A* = 3.67–5.83	0.57–0.65	NA	Bouzat et al. (1998)
*Tympanuchus phasianellus*	Minnesota	15	NA	0.43–1.0	185.6–498	Roy and Gregory (2019)
*Tetrao urogallus*	Eastern France	11	2.61	NA	15	Cayuela et al. (2019)
*Lyrurus tetrix*	Czech Republic	11	3.73–4.15	0.58–0.64	NA	Svobodová, Segelbacher, and Höglund (2011)
*Centrocercus minimus*	Colorado; Utah	22	2.29–4.29	0.49–0.60	NA	Zimmerman et al. (2019)
*Centrocercus urophasianus*	Western NA	15	*A* = 4.87–13.6	0.65–0.80	NA	Oyler‐McCance et al. (2022)

Abbreviations: *A*, mean number of alleles per locus, not corrected for sample size; *AR*, allelic richness corrected for sample size; *H*_O_, mean observed heterozygosity; NA, North America; *N*_e_, effective population size; NM, New Mexico; OK, Oklahoma; SOPR, shinnery oak prairie region of eastern New Mexico and western Texas.

The mean allelic richness (AR) for lek clusters in our study (mean *AR* range = 1.66–1.78) was lower than what has been previously reported for individuals sampled in Roosevelt County, New Mexico (AR = 5.53, Oyler‐McCance et al. 2016), but our calculation of *AR* was rarefied by the minimum sample size across lek clusters (Table 1). The mean *AR* for our entire dataset was 8.19, which was higher than previous results for the shinnery oak prairie region (mean *AR* = 7.83, Oyler‐McCance et al. 2016) and within the range for other ecoregions of the species' global distribution (mean *AR* range = 7.91–9.14, Oyler‐McCance et al. 2016). Although we did not detect genetic structure (discussed further below), the number of private alleles increased from our first two sampling periods (2002 and 2007–2010) to the final sampling period (2013–2014). This increase may be the result of greater sampling effort in period 3, an influx of migrants from nearby populations, or it may be an early indication of genetic differentiation that is not yet widely observable via other tests (Slatkin 1985).

Our relatedness analyses suggested that most individuals within each sampling period were not closely related to each other. Sampled individuals in 2013–2014 had more closely related pairs than earlier sampling periods (Supporting Information), but this could be due to more comprehensive sampling and thus greater likelihood that we sampled relatives that disperse from their natal origin. Some pairs of individuals were estimated to have high degrees of relatedness (i.e., parent‐offspring) despite being sampled at leks that are relatively distant from each other (e.g., leks 1 and 19). These observations provide support for continued gene flow even in areas where high‐traffic roadways and other anthropogenic infrastructure are present.

### 4.2 Population History and Effective Population Size

The extent to which small populations are affected by genetic drift and inbreeding depression is strongly influenced by the severity and duration of bottlenecks (Bortoluzzi et al. 2020). Such negative effects are more common for populations that experience recent and sudden demographic declines due to less time for purifying selection to act against deleterious variants (Ohta 1973; Marsden et al. 2016). Although some tests were consistent with a recent genetic bottleneck, the mode‐shift analysis and Sign test, and the apparent maintenance of genetic diversity between sampling periods in our study, provide support for the lack of a recent genetic bottleneck despite population fluctuations. Using 10 polymorphic loci for at least 30 individuals typically achieves a statistical power greater than 0.80 (Cornuet and Luikart 1996; Luikart and Cornuet 1998; Piry, Luikart, and Cornuet 1999). Our datasets included 12 polymorphic loci for groups of 48, 61, and 85 individuals, providing sufficient statistical power to detect a genetic bottleneck. It is important to note, however, that these tests are sensitive to violations of the assumptions of the underlying mutation models, which can affect the robustness of our conclusions (Peery et al. 2012). Nonetheless, we did see a consistent lack of bottlenecks across multiple test types and mutation models.

The effects of population declines on genetic diversity and differentiation may require more time to register than what the populations in our study have experienced thus far (Epps and Keyghobadi 2015; Gargiulo, Budde, and Heuertz 2024). Genetic bottlenecks may go undetected due to timelags, and genetic signals of bottlenecks can be obscured by immigration and the timing and duration of population bottlenecks (Cornuet and Luikart 1996; Peery et al. 2012). Furthermore, historical declines that reduce genetic diversity and precede recent bottlenecks can make it difficult to detect recent bottlenecks (Cornuet and Luikart 1996; Sonsthagen, Wilson, and Underwood 2017). Although these factors make inference from our bottleneck analyses more challenging, the maintenance of genetic diversity across our sampling periods provides additional support for the lack of a recent genetic bottleneck. Bottlenecks are considered to be detectable for 0.25–2.5 times 2*N*_e_ after the initiation of a population reduction (Peery et al. 2012); our sampling occurred well within these limits. Future research and conservation efforts that incorporate genetic analyses may first detect loss of genetic diversity in allelic richness, as a reduction in allelic variation is more commonly observed before a loss of heterozygosity following significant population declines (Maruyama and Fuerst 1985; Allendorf 1986).

Levels of genetic diversity are directly related to *N*_e_, and mating systems that have inherently skewed reproductive contribution can amplify stochastic genetic effects (Lande and Barrowclough 1987). The *N*_e_/*N* ratio for birds is typically 0.21 (Frankham 1995) but has been estimated as 0.585 for lesser prairie‐chickens (Garton et al. 2016). In our study, the estimated *N*_e_/*N* ratio was 0.19 (229.5/1155 = 0.19). Using the 0.21 ratio, the estimated population of 1155 lesser prairie‐chickens for the shinnery oak prairie region in 2014 (Nasman et al. 2022) should have an *N*_e_ of 242.55. This value falls within the ranges of our estimated 2013–2014 *N*_e_ values of 229.5 (95% CIs = 121.2–1023.1, *p*_crit_ = 0.05) and 349.1 (95% CIs = 176.4–2895.2, *p*_crit_ = 0.02). Using the 0.585 ratio calculation proposed by Garton et al. (2016), *N*_e_ is estimated at 676.26 (90% CIs = 197.7–1282.9), which is substantially higher but still within the 95% CIs of our estimates.

Although our estimates of *N*_e_ were not substantially different from expectations given the estimated population size, we recognize potential biases in these estimates. Sources of bias in estimating *N*_e_ include violating the assumption of no overlapping generations, unequal sex ratios, migration, and high variance in family sizes (Waples and Do 2010; Montarry et al. 2019). The infinite upper confidence interval for 2007–2010 may be due to reduced genetic drift when population abundance increases, which deteriorates the performance of the estimator (Gilbert and Whitlock 2015). Furthermore, demographic changes occurring over the course of our sampling period may introduce uncertainty and include additional error because the applied method of *N*_e_ estimation does not account for iteroparity, as is the case for lesser prairie‐chicken life history (Luikart et al. 2010; Waples, Antao, and Luikart 2014).

### 4.3 Lek Population Trends and Genetic Diversity

Our findings align with studies of other species (e.g., small mammals) with cyclical population fluctuations, which find that these cycles have limited effects on measures of genetic diversity, despite theoretical predictions to the contrary (Pilot et al. 2010; McEachern et al. 2011; Rikalainen et al. 2012). Our analyses of genetic diversity across different sampling periods did not reveal significant differences related to varying levels of lesser prairie‐chicken abundance. Populations with significant fluctuations may maintain genetic diversity through increased migration rates during high‐density periods, mitigating losses during low densities (Pilot et al. 2010; Rikalainen et al. 2012), and returning to pre‐bottleneck levels given sufficient movement of individuals among subpopulations (McEachern et al. 2011; Gauffre et al. 2014). Short‐ and long‐distance movements of males and females among leks (Lawrence et al. 2022) would promote genetic homogenization if these movements result in successful reproduction.

A greater distribution of sampled leks, more individuals sampled per lek, and/or the use of molecular markers such as single nucleotide polymorphisms (SNPs) could improve our confidence in examining genetic diversity among leks (Zimmerman, Aldridge, and Oyler‐McCance 2020). Furthermore, immigration may reduce differential regional densities, such that high levels of immigration can synchronize population dynamics and counteract potential genetic differentiation (Franklin, Myers, and Cory 2014).

### 4.4 Population Structure

We evaluated population structure using multiple methods to provide strong evidence for a lack of genetic differentiation for lesser prairie‐chickens in eastern New Mexico and between sampling periods. Although some leks in this study are separated by substantial distances and by landscape features that are avoided by lesser prairie‐chickens, such as mesquite and anthropogenic infrastructure (Boggie et al. 2017; Falkowski et al. 2017; Plumb et al. 2019; Lawrence et al. 2022), the lack of genetic differentiation we observed provides further evidence for little genetic change across sampling periods and suggests that gene flow may be occurring at the landscape level.

It is possible that lesser prairie‐chicken migration rates are sufficiently high to prevent significant differentiation via genetic drift. Effects of isolation on gene flow, however, may take more time to register (Landguth et al. 2010). Male lesser prairie‐chicken natal dispersal is generally within a few kilometers, while long‐distance dispersal movements for females are more common, with some individuals dispersing up to 71 km (Pitman et al. 2006; Earl et al. 2016). The cumulative effects of short‐range dispersals may translate to long‐range connectivity. Furthermore, it is possible for density‐dependent behavioral factors caused by habitat loss and population contraction to induce individuals to disperse, which could introduce rare alleles to other populations (Cornuet and Luikart 1996; Kvistad et al. 2015).

Relatively few studies have examined lesser prairie‐chicken genetic structure. Oyler‐McCance et al.'s (2016) range‐wide analysis did not find structure within the shinnery oak prairie ecoregion but did detect structuring between the four ecoregions across the species' range in New Mexico, Texas, Oklahoma, Colorado, and Kansas. Of all the regions we sampled, lek clusters 1 and 2 (Figure S1) were the most isolated from other leks in our study area. We expected that these lek clusters were more likely to be genetically differentiated from others due to large geographic distances and potential barriers (i.e., a high‐traffic highway). There was some support for this prediction in the pairwise *F*_ST_ comparisons (Figure 3; Figure S2), but not in other clustering analyses. These leks were represented by relatively few individuals, and low sample sizes may have reduced our ability to detect genetic structure, which would also have implications for the design and interpretation of our other genetic analyses (e.g., effective population size estimation).

Lesser prairie‐chickens in New Mexico appear to have maintained their genetic diversity despite population declines and fluctuations. Populations on the fringe of a species' distribution are generally expected to exhibit lower genetic diversity (Soule 1973; Hoffmann and Blows 1994; Lesica and Allendorf 1995), but our results are comparable to those in the core of the species' range in Kansas (Oyler‐McCance et al. 2016). This similarity could be due to adaptations in behavior and natural history that reduce the genetic consequences of being a peripheral population (Eckert, Samis, and Lougheed 2008). Regardless of current genetic diversity levels, persistent threats to lesser prairie‐chicken populations mean that continued monitoring of genetic diversity and efforts to increase available habitat and *N*_e_ should improve the likelihood of the species' long‐term survival.

## 5 Conservation Implications

Current lesser prairie‐chicken populations in the southern portion of their range have low population sizes and are at high risk of extirpation due to extended drought and anthropogenic alteration of habitat (USFWS 2021; Nasman et al. 2022). Increased efforts to restore habitat and decrease fragmentation are essential to increasing *N*_e_ (Hagen and Giesen 2020; USFWS 2021). Due to natural and anthropogenically influenced fluctuations in lesser prairie‐chicken populations, high levels of migration among populations may be necessary to maintain genetic diversity and *N*_e_ (Mills and Allendorf 1996; Vucetich and Waite 2000). Minimum population sizes of 100 and 1000 are considered necessary to limit losses in total fitness and conserve evolutionary potential, respectively (Frankham, Bradshaw, and Brook 2014).

Overall, the factors influencing genetic diversity are complex and can require significant effort to evaluate. Maintaining sufficient genetic diversity to allow for adaptation to shifts in climate, habitat composition and structure, and pathogens is essential to a species' persistence (Van Oppen et al. 2015). Conserving lesser prairie‐chicken populations at the periphery of the species' range in New Mexico, where conditions are drier and warmer than northern regions of its range, may preserve adaptive genes that are important for the species' persistence in light of current climate challenges and future climate projections (Ross et al. 2016; Williams, Cook, and Smerdon 2022). Efforts that maintain or improve gene flow and genetic diversity, such as increasing available habitat and population connectivity, will likely increase the probability for the lesser prairie‐chicken to persist as environmental conditions change.

## Author Contributions

**Andrew J. Lawrence:** conceptualization (lead), data curation (lead), formal analysis (lead), investigation (lead), methodology (lead), project administration (lead), software (lead), supervision (equal), validation (lead), visualization (lead), writing – original draft (lead), writing – review and editing (equal). **Scott A. Carleton:** funding acquisition (lead), project administration (supporting), resources (supporting), supervision (supporting), writing – review and editing (supporting). **Sara J. Oyler‐McCance:** data curation (supporting), resources (supporting), writing – review and editing (equal). **Randy W. DeYoung:** data curation (supporting), resources (supporting), writing – review and editing (supporting). **Clay T. Nichols:** funding acquisition (equal), writing – review and editing (supporting). **Timothy F. Wright:** conceptualization (supporting), data curation (supporting), formal analysis (supporting), investigation (supporting), methodology (supporting), project administration (supporting), supervision (equal), validation (supporting), writing – review and editing (equal).

## Disclosure

Any use of trade, firm, or product names is for descriptive purposes only and does not imply endorsement by the U.S. Government. The findings and conclusions in this article are those of the author(s) and do not necessarily represent the views of the U.S. Fish and Wildlife Service.

## Conflicts of Interest

All authors agree with the contents of the manuscript. Any research in the manuscript not carried out by the authors has been fully acknowledged, and funding and author benefits have been declared. All methods used in this study were approved by the New Mexico State University IACUC committee. The authors declare no conflicts of interest.

### Open Research Badges

This article has earned an Open Data badge for making publicly available the digitally‐shareable data necessary to reproduce the reported results. The datasets and code to run analyses are available on Dryad at https://doi.org/10.5061/dryad.8931zcrxt.

## Supporting information


Data S1.

## Data Availability

Datasets, R code, and supplementary materials available on Dryad at https://doi.org/10.5061/dryad.8931zcrxt.
