# Supplementary material for: Maintenance of Genetic Diversity Despite Population Fluctuations in the Lesser Prairie‐Chicken (Tympanuchus pallidicinctus)
Source: Ecol Evol. 2025 Jan 23;15(1):e70879. doi: 10.1002/ece3.70879 (PMC11757004; doi:10.1002/ece3.70879)
Supplement: Supplementary file 1 — Data S1. [file ECE3-15-e70879-s001.docx]

# SUPPLEMENTARY MATERIALS

**TITLE:** Maintenance of genetic diversity despite population fluctuations in the lesser prairie-chicken (*Tympanuchus pallidicinctus*)

Andrew J. Lawrence^1^, Scott A. Carleton ^2^, Sara J. Oyler-McCance^3^, Randy W. DeYoung^4^, Clay T. Nichols^5^, Timothy F. Wright^1^

^1^Department of Biology, New Mexico State University, Las Cruces, New Mexico, USA

^2^Division of International Conservation, International Affairs, U.S. Fish and Wildlife Service, Falls Church, VA, USA

^3^U.S. Geological Survey, Fort Collins Science Center, Fort Collins, Colorado, USA

^4^Caesar Kleberg Wildlife Research Institute, Texas A&M University-Kingsville, Kingsville, Texas, USA

^5^Ecological Services, United States Fish and Wildlife Service, Albuquerque, New Mexico, USA

Corresponding author: Andrew Lawrence; email: Andrew.lawrence@colostate.edu

Andrew J. Lawrence ORCID: 0000-0001-5132-1961

Scott A. Carleton ORCID: 0009-0006-4702-3973

Sara J. Oyler-McCance ORCID: 0000-0003-1599-8769

Timothy F. Wright ORCID: 0000-0003-2859-5360

Corresponding author: Andrew Lawrence; email: [Andrew.lawrence@colostate.edu](mailto:Andrew.lawrence@colostate.edu)

Any use of trade, firm, or product names is for descriptive purposes only and does not imply endorsement by the U.S. Government.

| Table S1. Evanno method parameter results based on 10 iterations of *K* ranging 1 to 10 for lesser prairie-chicken population structure in 2002 and 2013 – 2014 in Chaves, Lea, and Roosevelt Counties, New Mexico, and from 2007 – 2010 in Bailey, Cochran, and Yoakum Counties, Texas, USA. The Evanno method cannot find the best *K* if *K* = 1, which is supported by our additional analyses. | | | | | | | | | |
| --- | --- | --- | --- | --- | --- | --- | --- | --- | --- |
| **# K** | **Reps** | **Mean LnP(*K*)** | **StDev LnP(*K*)** | **Ln'(*K*)** | | **\|Ln''(K)\|** | | **Delta *K*** | |
| 1 | 10 | -7891.16 | 0.36 | NA | NA | | NA | |  |
| 2 | 10 | -8174.09 | 32.15 | -282.93 | 322.54 | | 10.03 | |  |
| 3 | 10 | -8779.56 | 194.35 | -605.47 | 260.33 | | 1.34 | |  |
| 4 | 10 | -9645.36 | 696.71 | -865.8 | 369.34 | | 0.53 | |  |
| 5 | 10 | -10141.77 | 895.56 | -496.41 | 298.01 | | 0.33 | |  |
| 6 | 10 | -10936.19 | 602.42 | -794.42 | 433.9 | | 0.72 | |  |
| 7 | 10 | -11296.71 | 1056.74 | -360.52 | 658.61 | | 0.62 | |  |
| 8 | 10 | -10998.62 | 763.21 | 298.1 | 1011.7 | | 1.32 | |  |
| 9 | 10 | -11712.27 | 1348.92 | -713.65 | 893.02 | | 0.66 | |  |
| 10 | 10 | -11532.9 | 1550.97 | 179.37 | NA | | NA | |  |

| Table S2. Mean observed heterozygosity (*H_O_*) and expected heterozygosity (*H_e_*) per site, mean allelic richness across loci (*AR*), and total number sampled individuals and private alleles for each locality (refer to Fig. S1) in 2013 – 2014 in Chaves, Lea, and Roosevelt Counties, New Mexico, USA.  *H_O_*, *H_e_*, and *AR* were calculated across all loci. We report the standard deviation in parentheses, and the observed values for each site that sampled single individuals when means were not possible. Overall mean *H_O_*, *H_e_*, and *AR* were calculated for the entire sampled population. | | | | | | |
| --- | --- | --- | --- | --- | --- | --- |
| **Locality** | **Mean *H*_O_** | **Mean *H*_e_** | **Mean *AR*** | **Private alleles** | **Sample size** |  |
| 1 | 0.67 (0.38) | 0.70 (0.31) | 1.71 (0.27) | - | 2 |  |
| 2 | 0.75 (0.39) | 0.64 (0.37) | 1.69 (0.37) | 1 | 2 |  |
| 3 | 0.83 (0.24) | 0.82 (0.19) | 1.83 (0.10) | - | 2 |  |
| 4 | 0.69 (0.30) | 0.77 (0.19) | 1.71 (0.28) | - | 3 |  |
| 5 | 0.67 (NA) | 0.33 (NA) | 1.67 (NA) | - | 1 |  |
| 6 | 0.75 (0.20) | 0.76 (0.10) | 1.76 (0.11) | - | 7 |  |
| 7 | 0.71 (0.24) | 0.83 (0.13) | 1.80 (0.10) | - | 3 |  |
| 8 | 1.00 (NA) | 0.5 (NA) | 2.00 (NA) | - | 1 |  |
| 9 | 0.78 (0.22) | 0.76 (0.13) | 1.76 (0.13) | - | 8 |  |
| 10 | 0.73 (NA) | 0.5 (NA) | 1.58 (NA) | - | 1 |  |
| 11 | 0.91 (NA) | 0.5 (NA) | 1.75 (NA) | - | 1 |  |
| 12 | 0.75 (0.26) | 0.81 (0.18) | 1.79 (0.14) | - | 2 |  |
| 13 | 0.71 (0.25) | 0.77 (0.24) | 1.75 (0.23) | 1 | 2 |  |
| 14 | 0.77 (0.24) | 0.74 (0.20) | 1.74 (0.20) | - | 4 |  |
| 15 | 0.78 (0.30) | 0.74 (0.26) | 1.74 (0.26) | 2 | 5 |  |
| 16 | 0.71 (0.33) | 0.71 (0.23) | 1.71 (0.16) | - | 2 |  |
| 17 | 0.91 (NA) | 0.5 (NA) | 1.75 (NA) | - | 1 |  |
| 18 | 0.75 (NA) | 0.38 (NA) | 1.75 (0.45) | 1 | 1 |  |
| 19 | 0.72 (0.31) | 0.75 (0.16) | 1.74 (0.17) | - | 3 |  |
| 20 | 0.76 (0.15) | 0.73 (0.17) | 1.74 (0.16) | - | 5 |  |
| 21 | 0.61 (0.37) | 0.73 (0.22) | 1.66 (0.28) | - | 3 |  |
| 22 | 0.78 (0.29) | 0.83 (0.08) | 1.82 (0.11) | - | 3 |  |
| 23 | 0.78 (0.19) | 0.77 (0.13) | 1.77 (0.13) | 1 | 7 |  |
| 24 | 0.83 (0.19) | 0.75 (0.15) | 1.75 (0.15) | 4 | 7 |  |
| 25 | 0.73 (0.29) | 0.79 (0.14) | 1.78 (0.13) | - | 4 |  |
| 26 | 0.58 (NA) | 0.33 (NA) | 1.58 (0.51) | - | 1 |  |
| 27 | 0.65 (0.26) | 0.65 (0.11) | 1.76 (0.11) | - | 4 |  |
| **Overall** | **0.75 (0.12)** | **0.76 (0.11)** | **8.19 (3.49)** | **-** | **85** |  |


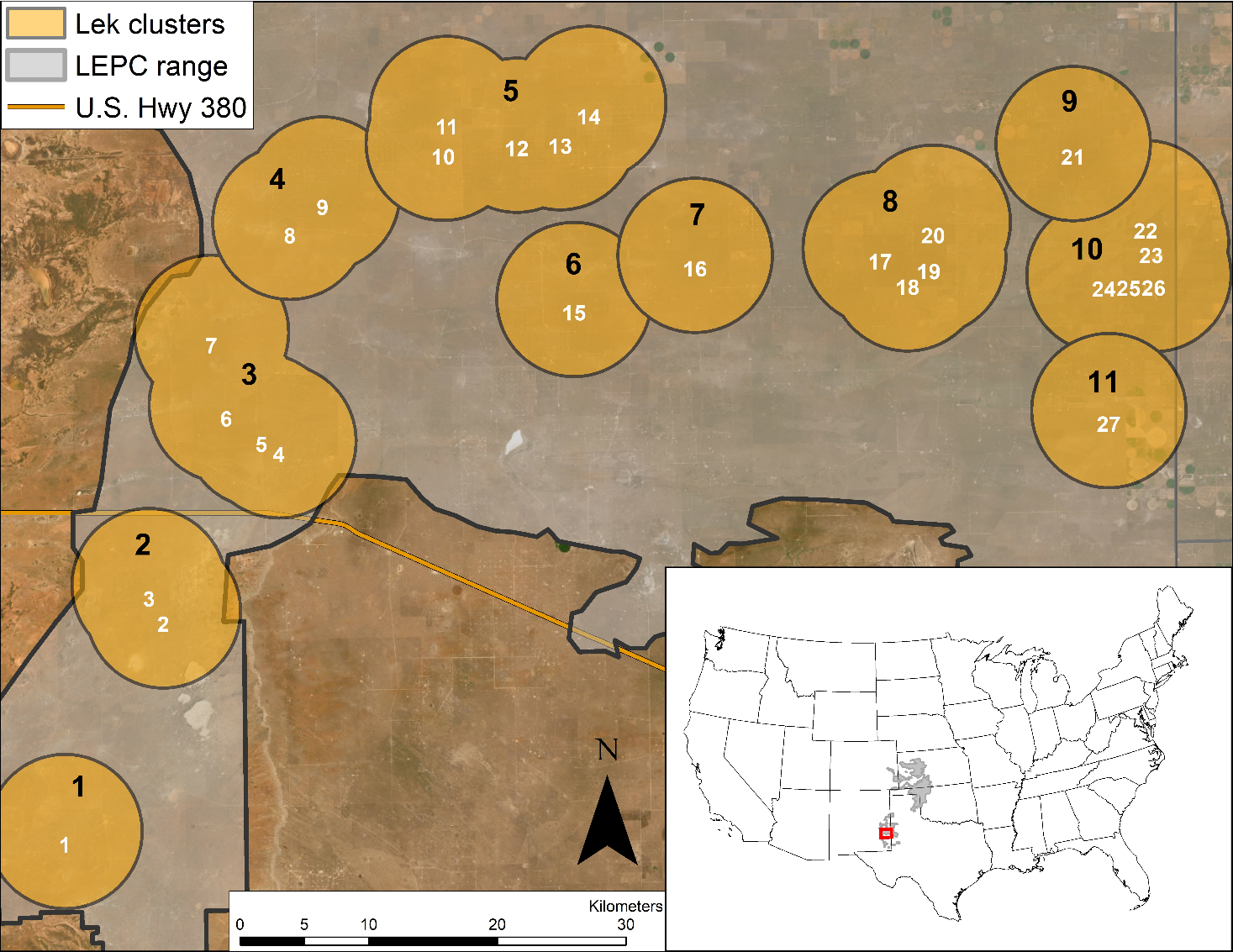


**Fig. S1** Locations of lesser prairie-chicken (LEPC) leks and DNA collection sites (numbered 1 – 27 in white) in 2013 – 2014 in Chaves, Lea, and Roosevelt Counties, New Mexico, USA. Lek clusters (orange) are numbered in black. The range of the species is shaded in gray. The sampling area focused on the core of the species’ extant distribution in New Mexico.

**
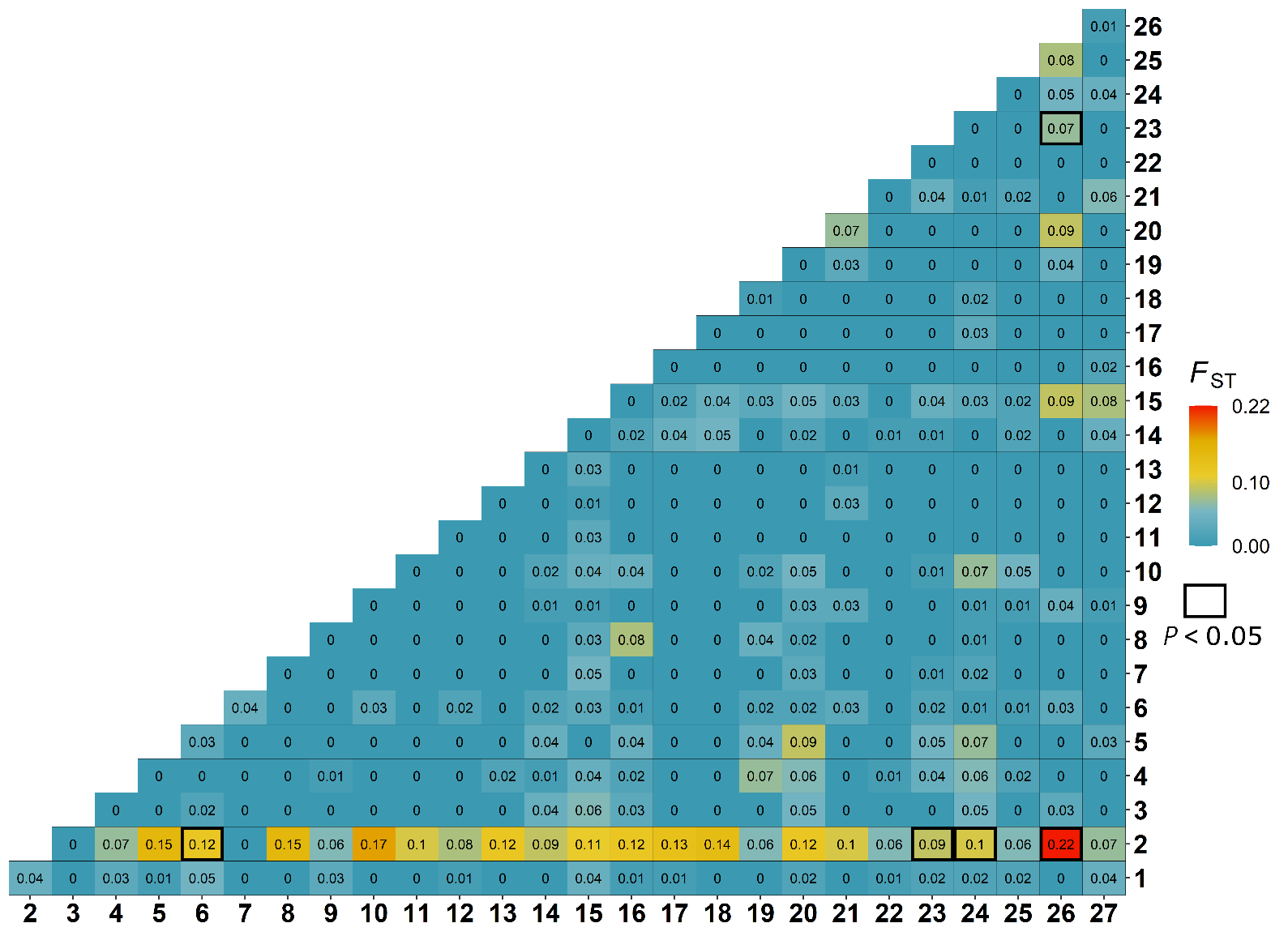
**

**Fig. S2** Pairwise population matrix of *F_ST_* values (Weir and Cockerham 1984) for lesser prairie-chicken leks in Chaves, Lea, and Roosevelt Counties, New Mexico, USA 2013 – 2014. Corresponding lek identification numbers are available in Fig. S1.


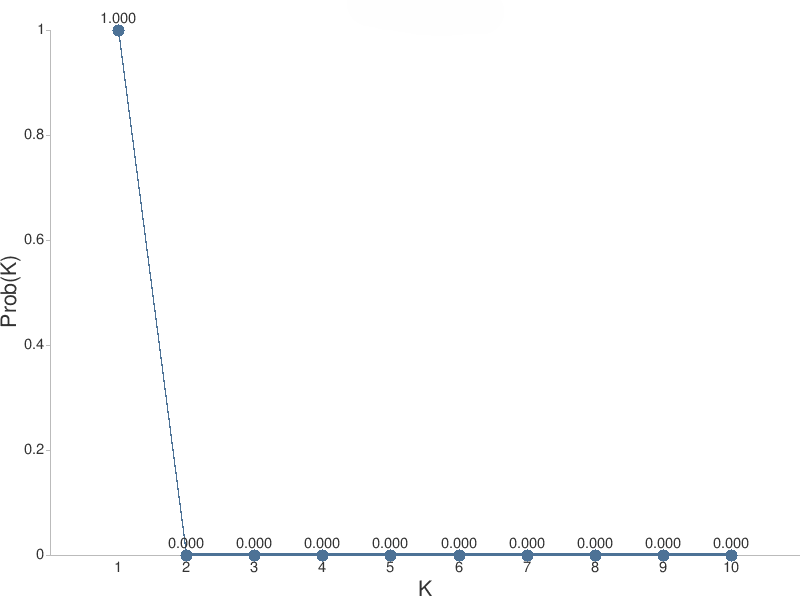


### **Fig. S3** Estimated probability value for each of the number of lesser prairie-chicken populations (*K*) estimated by program STRUCTURE in 2002 and 2013 – 2014 in Chaves, Lea, and Roosevelt Counties, New Mexico, and from 2007 – 2010 in Bailey, Cochran, and Yoakum Counties, Texas, USA. The maximum value is observed at *K* = 1.


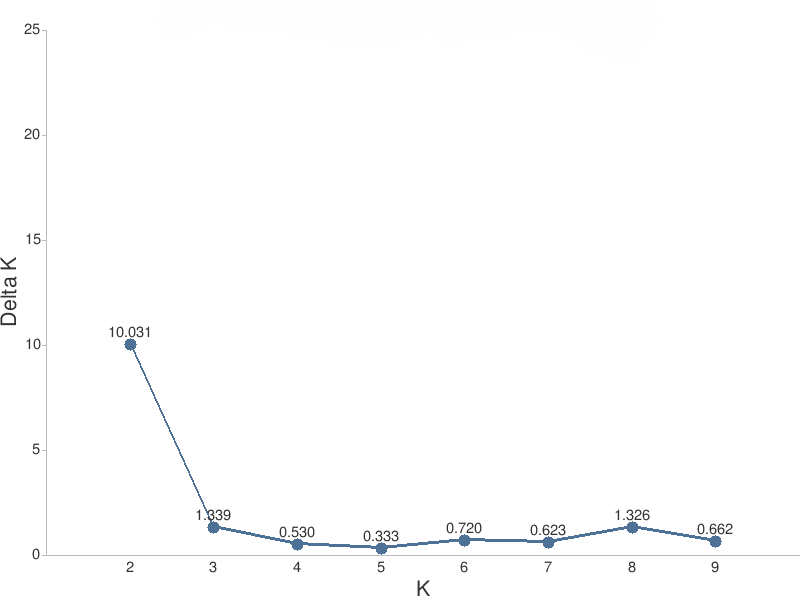


### **Fig. S4** Estimated number of lesser prairie-chicken populations (*K*) estimated using Delta *K* (Evanno method) in 2002 and 2013 – 2014 in Chaves, Lea, and Roosevelt Counties, New Mexico, and from 2007 – 2010 in Bailey, Cochran, and Yoakum Counties, Texas USA. This method cannot find the best *K* if *K* = 1, which is the likely *K* supported by additional structure analyses.

###

***
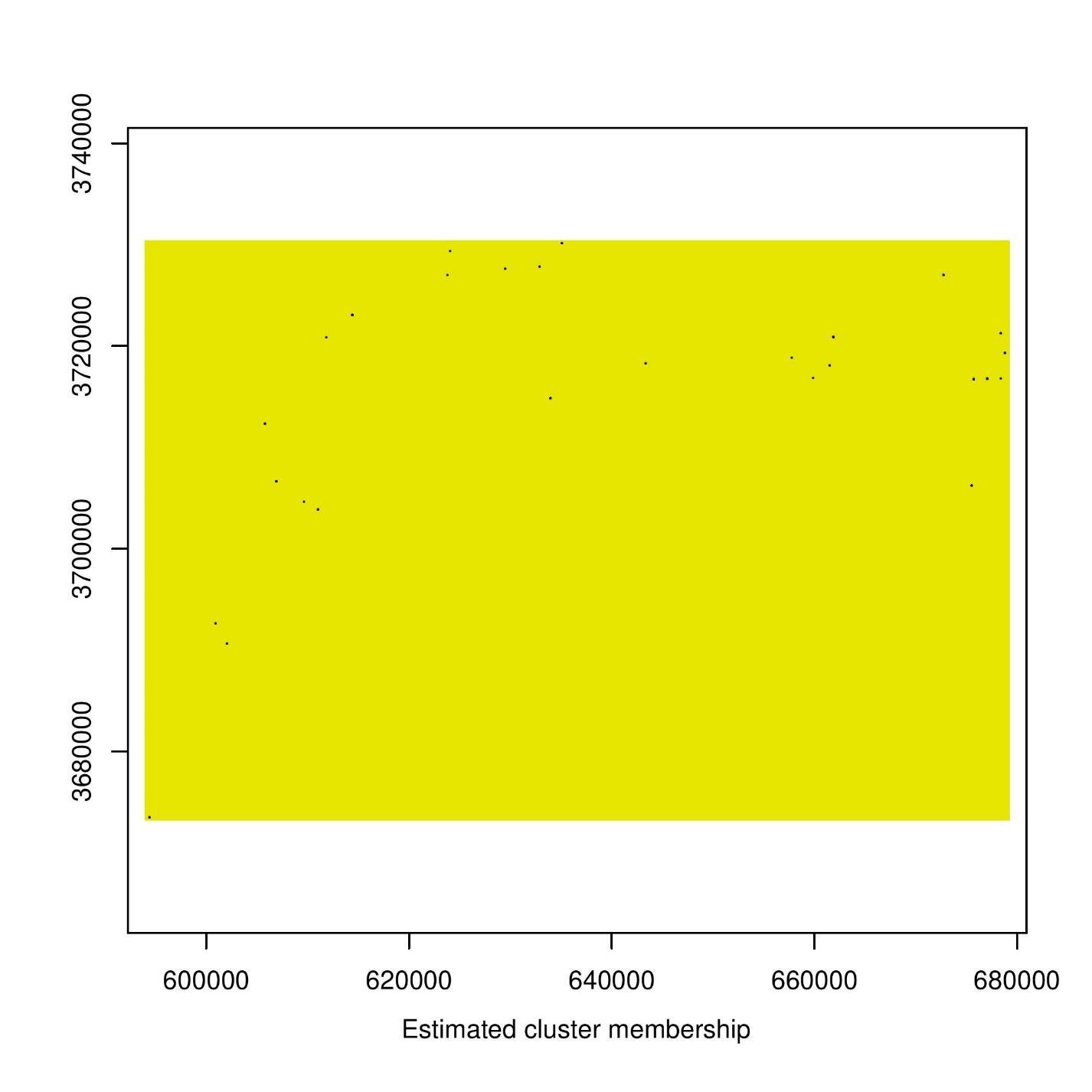
***

### **Fig. S5** Geneland population structure results estimating a single population for lesser prairie-chickens in Chaves, Lea, and Roosevelt Counties, New Mexico, USA 2013 – 2014. Both the correlated and uncorrelated Geneland models estimated a single population.

# LITERATURE CITED

Weir BS, Cockerham CC. 1984. Estimating F-Statistics for the Analysis of Population Structure. Evolution 38:1358–1370.
